# Bleb Point: Mimicker of Pneumothorax in Bullous Lung Disease

**DOI:** 10.5811/westjem.2015.3.24809

**Published:** 2015-04-09

**Authors:** Christopher Gelabert, Mathew Nelson

**Affiliations:** North Shore University Hospital, Department of Emergency Medicine, Division of Emergency Ultrasound, Manhasset, New York

## Abstract

In patients presenting with severe dyspnea, several diagnostic challenges arise in distinguishing the diagnosis of pneumothorax versus several other pulmonary etiologies like bullous lung disease, pneumonia, interstitial lung disease, and acute respiratory distress syndrome. Distinguishing between large pulmonary bullae and pneumothorax is of the utmost importance, as the acute management is very different. While multiple imaging modalities are available, plain radiographs may be inadequate to make the diagnosis and other advanced imaging may be difficult to obtain. Ultrasound has a very high specificity for pneumothorax. We present a case where a large pulmonary bleb mimics the lung point and therefore inaccurately suggests pneumothorax.

## INTRODUCTION

Bullous lung disease is a spectrum of disease with multiple causes, most commonly smoking.^1^ These giant bullae develop and can progress to occupy much of the hemithorax and compress surrounding normal lung parenchyma.^2^–^4^ A pneumothorax is a collection of air in the pleural space with subsequent lung collapse. Plain films will classically demonstrate a white linear density (pleura) outlining a distinct area of black pleural space where lung markings are absent.^5^ Because of these similarities, it can be difficult to differentiate bullae from pneumothoraces.

Lung ultrasound is based on the interpretation of several artifacts. The first important sign to be checked is lung sliding. It is horizontal movement of the pleural line during active and passive inspiration. The two pleural layers are not distinct sonographically, thus the sliding is an indirect sign indicating the presence of the visceral pleura adhering to the parietal pleura. Lung sliding can be represented on M-Mode by a granular pattern below the pleural line, often described as “sea shore sign” or “sand on a beach.” Presence of lung sliding rules out pneumothorax with 100% specificity.^6^

Absence of lung sliding can be a result of pneumothorax, massive atelectasis, main-stem intubation, pulmonary contusion, acute respiratory distress syndrome, and pleural adhesions.^6^ Since absence of lung sliding alone may not be enough to diagnose pneumothorax, confirmation can be achieved by gradually moving the probe inferiorly on the chest wall, targeted at the detection of a point on the chest wall where a respiratory pattern (i.e., lung sliding) is visualized again and intermittently replaces the motionless pleura. This point is named the “lung point” and has been described as having 100% specificity for detection of pneumothorax.^7^

We present a case report of a patient with severe bullous lung disease in respiratory distress and sonographic findings suggestive of pneumothorax.

## CASE REPORT

**Figure 1:**
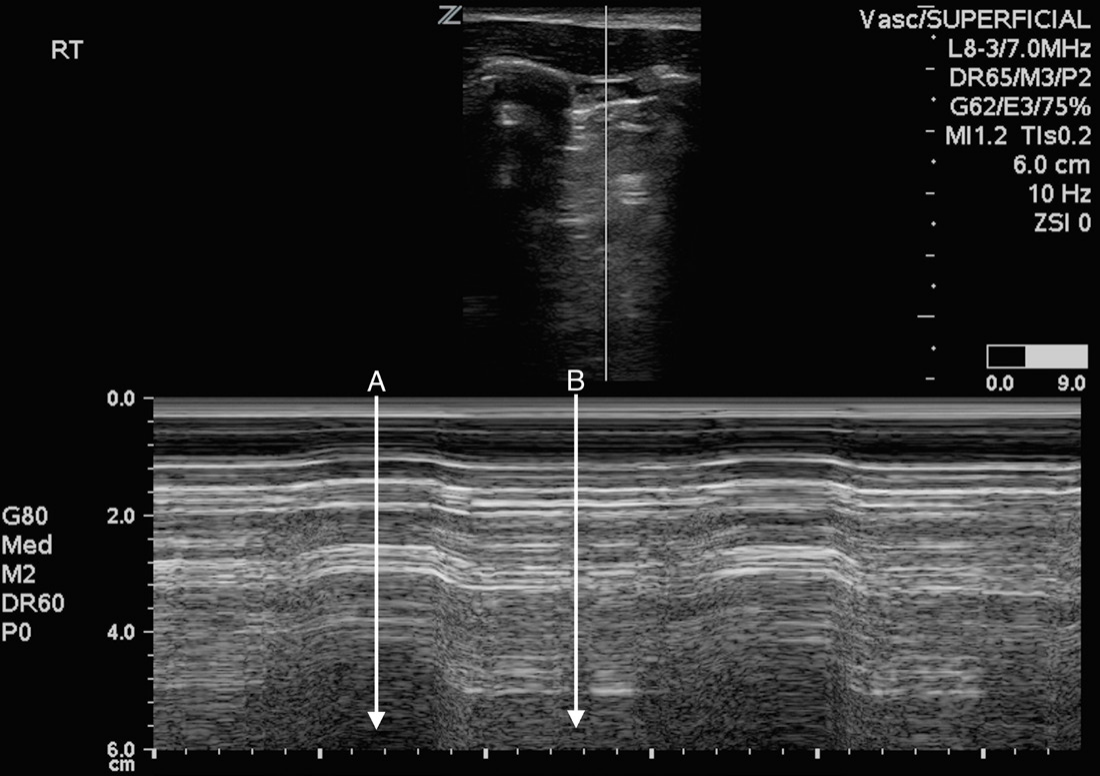
M-mode ultrasound of the right lung, demonstrating bleb point. A. No lung sliding (barcode sign). B. Lung sliding (seashore sign).

A 33-year-old male with severe chronic obstructive pulmonary disease and unexplained extensive bilateral bullous emphysema presented to the emergency department with a chief complaint of dyspnea. The patient was in moderate respiratory distress with vital signs upon presentation: blood pressure 130/84mmHg, pulse 99, respiratory rate 32, pulse oximetry 94% on 4L nasal cannula, temperature 37°C. That morning he developed markedly worsening dyspnea, despite supplemental home oxygen therapy. He also reported subjective fevers and cough. Physical exam demonstrated respiratory distress, with coarse upper breath sounds and diminished breath sounds at the bases bilaterally. He was placed on high-flow nasal cannula due to worsening respiratory distress. Portable chest radiograph demonstrated large bullous emphysema on the right lung with complete obliteration of normal lung and possible pneumothorax. The patient was unable to lie flat for computed tomography (CT), so bedside ultrasound (Figure 1 and Video) was subsequently performed using a high frequency linear transducer, demonstrating normal lung sliding at the left apex. No lung sliding was noted at the right apex and lung point was also noted. Differential diagnoses included pneumothorax, worsening bullous emphysema, and pneumonia.

**Figure 2:**
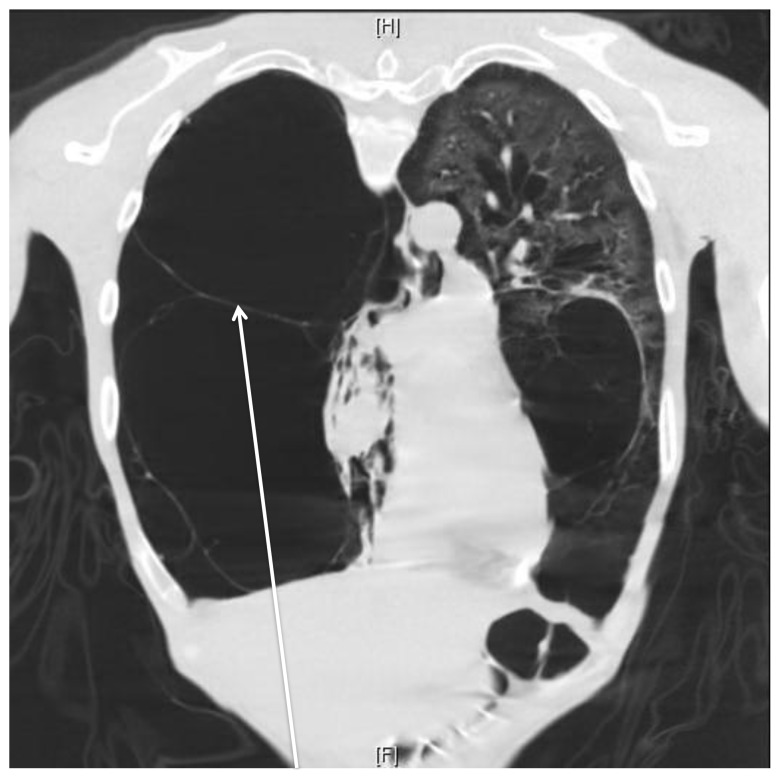
Coronal chest computed tomography demonstrating extensive bullous lung disease and no pneumothorax (arrow).

The patient rapidly improved with oxygen, nebulized albuterol and ipratropium, intravenous methylprednisolone, and antibiotics, so tube thoracostomy was held. He became stable enough for CT (Figure 2), which demonstrated complete collapse of the right lung secondary to extensive progressive bullous emphysema with extensive bilateral bullae and bronchiectasis. There were air-fluid levels at the right lung base concerning for superinfection vs. secretions. No pneumothorax was appreciated. The patient was admitted to the respiratory stepdown unit, where steroids and antibiotics were continued, and the patient was subsequently transferred to a specialty tertiary hospital for lung transplant.

## DISCUSSION

Distinguishing pneumothorax from bullous emphysema is a difficult but important distinction in management of the severely dyspneic patient. Patients with bullous emphysema, especially large bullae are at higher risk for pneumothorax.^5^ Thus, risk factors and often clinical exam are less than helpful. Frequently chest radiograph is unable to differentiate bullous emphysema from pneumothorax, but chest CT, the gold standard, is often difficult for patients to tolerate. A physician may also feel that the patient is not stable enough to go to radiology for a CT. This creates a dilemma as to what diagnostic test will aid in the accurate assessment of these acutely ill patients.

Lung ultrasound has been proven to be valuable in assessing pneumothorax in the unstable patient, especially compared to portable chest radiograph.^8^,^9^ While there has been some argument about the sonographic appearance of bullous emphysema, anecdotal reports and case series have determined that ultrasound is still able to differentiate bullous emphysema from pneumothoraces.^10^,^11^ Presence of lung sliding effectively rules out pneumothorax despite concomitant lung disease while presence of a lung point was previously thought to effectively rule in pneumothorax.

However, none of those cases involved discovery of a bleb mimicking a lung point, or “bleb point.” We postulate that because of the severity of bullous emphysema that the amount of healthy lung tissue was minimal and that the visceral pleura was so thin at the junction of parietal pleura that M-Mode ultrasound was unable to detect any sliding. Further study is required to examine the utility of these findings in larger populations.

## Figures and Tables

**Video:** Ultrasound demonstrating normal lung sliding in right apex and bleb point.
